# Anti‐inflammatory and Gastroprotective Effects of the *Chamaerops humilis* L. (DOUM) Fruit Aqueous Extract in Experimental Models of Edema and Gastric Ulcer

**DOI:** 10.1002/cbdv.202403241

**Published:** 2025-04-03

**Authors:** Yasmina Jaouhari, Hamid Kabdy, Ouijdane El hatimy, Hajar Azraida, Baslam Abdelmounaim, Abdelfattah Aitbaba, Laadraoui Jawad, Yassine Chait, Rachida Aboufatima, Soad Moubtakir, Loubna El Yazouli, Stefania Garzoli, Abderrahman Chait

**Affiliations:** ^1^ Department of Biology, Faculty of Sciences Semlalia Laboratory of Pharmacology, Neurobiology, Anthropobiology and Environment, Cadi Ayyad University Marrakech Morocco; ^2^ Laboratory of Physiopathology, Genetic Molecular and Biotechnology, Faculty of Sciences, Aïn Chock, Hassan II University Casablanca Morocco; ^3^ Agadir Souss Massa University Hospital, Faculty of Medicine and Pharmacy, Ibn Zohr University Agadir Morocco; ^4^ Laboratory of Biological Engineering, Faculty of Sciences and Technology, Sultan Moulay Slimane University Beni Mellal Morocco; ^5^ Department of Chemistry and Technologies of Drug Sapienza University Rome Italy

**Keywords:** anti‐inflammatory, *Chamaerops humilis*, gastric ulcers, gastroprotective, natural remedies, traditional medicine

## Abstract

Chronic inflammation can lead to various diseases, including gastric ulcers, highlighting the need for effective therapeutic strategies. The traditional use of *Chamaerops humilis* in Moroccan folk medicine for treating gastrointestinal disorders underscores its potential as a valuable natural remedy. This study rigorously evaluates the anti‐inflammatory and gastroprotective effects of the aqueous extract of *C. humilis* (AECH) through a series of well‐established animal models, including carrageenan‐induced paw edema, xylene‐induced ear edema, and ethanol/hydrochloric acid (EtOH/HCL)‐induced gastric ulcers. The results reveal that AECH significantly reduces inflammation and ulcer severity in a dose‐dependent manner, demonstrating potent efficacy in decreasing paw and ear edema while markedly mitigating ulceration induced by EtOH/HCL exposure. Notably, AECH achieved up to 100% inhibition of ulcer lesions at higher doses. These findings not only validate the traditional applications of *C. humilis* but also highlight its promising role as a natural therapeutic agent for managing inflammatory conditions and gastric ulcers.

## Graphical abstract

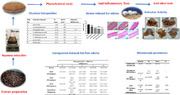

## 1 Introduction

Inflammation is the body's natural defense mechanism, involving a coordinated response by blood vessels, immune cells, and chemical mediators to isolate and repair damaged tissues [1]. The acute inflammatory response is designed to eliminate harmful stimuli and restore normal physiological conditions through processes such as phagocytosis, apoptosis, and the activation of proinflammatory mediators [2]. However, when this protective mechanism becomes chronic, it can contribute to various diseases [3] including peptic ulcer disease (PUD) [4].

PUD, characterized by erosions in the stomach or duodenal lining, is a significant global health issue affecting millions of people worldwide [5]. A key contributor to PUD is the prolonged use of nonsteroidal anti‐inflammatory drugs (NSAIDs), which are commonly prescribed for pain and inflammation management. NSAIDs, such as ibuprofen, aspirin, and naproxen, work by inhibiting cyclooxygenase (COX) enzymes, which play a critical role in the production of prostaglandin compounds involved in the inflammatory process [4]. While this mechanism effectively reduces inflammation, it also reduces the production of protective prostaglandins in the stomach lining, leading to increased vulnerability to gastric acid [4].

The widespread use of NSAIDs has led to a growing concern about their side effects, particularly in long‐term treatments for chronic inflammatory conditions like arthritis and other musculoskeletal disorders [4]. Previous studies have shown that prolonged NSAID use is a leading cause of drug‐induced peptic ulcers and related complications, with an estimated 15%–30% of chronic NSAID users experiencing significant gastrointestinal side effects [5]. These complications highlight the urgent need for safer therapeutic alternatives that can manage inflammation without causing harm to the gastrointestinal system [6].

Traditional medicine has offered a rich tapestry of remedies for various ailments, and plants have been a primary source of therapeutic agents for centuries [7]. One such plant, *Chamaerops humilis* L. (Arecaceae), known as Doum palm in Morocco, has a long history of traditional use for treating numerous conditions [8]. The fruits and roots of this plant have been particularly valued for their purported benefits in gastrointestinal disorders, including constipation, intestinal worms, and diarrhea [9]. Additionally, the plant possesses antibacterial, antitumoral, and antioxidant properties [10, 11]. However, despite this traditional use, there is a paucity of scientific research exploring the therapeutic potential of *C. humilis*, particularly regarding its anti‐ulcer and anti‐inflammatory properties.

This study aims to bridge this knowledge gap by investigating the gastroprotective effects of the aqueous extract from the fruits of *C. humilis L* (AECH) against ethanol/hydrochloric acid (EtOH/HCL)‐induced gastric ulcers in rats, as well as its anti‐inflammatory effects using the carrageenan‐induced paw edema model in rats and the xylene‐induced ear edema model in mice. The outcomes of this research could provide scientific validation for the traditional use of Doum palm and potentially lead to the development of new, natural therapeutic agents for managing gastrointestinal and inflammatory disorders.

## 2 Results

### 2.1 Phytochemical Characterization by Liquid Chromatography‐Tandem Mass Spectrometry

The aqueous extract of *C. humilis* (AECH) was analyzed using high‐performance liquid chromatography‐photodiode array‐tandem mass spectrometry (HPLC–PDA–MS/MS), allowing for the identification of major phytoconstituents through their MS/MS fragments and retention times (Rts) (Table 1). This analysis revealed eight primary secondary metabolites, predominantly phenolic acids. The results, detailed in Table 2, show that quinic acid, chlorogenic acid derivatives, and *p*‐coumaric and ferulic acids are the main components of this extract. Among these compounds, quinic acid (Rt = 1.60 min) was identified as an early eluting metabolite, while chlorogenic acid (Rt = 10.10 min) and its derivatives, such as 3‐caffeoylquinic acid (Rt = 14.12 min) and 5‐caffeoylquinic acid (Rt = 19.10 min), were detected later in the separation, consistent with their structural diversity and contribution to the extract's antioxidant properties. Additionally, ferulic acid (Rt = 24.10 min) and its derivative feruloylquinic acid (Rt = 15.29 min).

**TABLE 1:** Annotated compounds from *C. humilis* (AECH) aqueous extract using liquid chromatography‐tandem mass spectrometry (LC‐MS/MS).

Peak No.	Proposed compound	Rt (min)	[M‐H]—(*m/z*)	Fragments MS/MS (*m/z*)
1	Quinic acid	1.60	191	—
2	Cinnamoyl glucose	8.22	309	—
3	Chlorogenic acid	10.10	353	179
4	3‐Caffeoylquinic acid	14.12	359	—
5	Feruloylquinic acid	15.29	362	—
6	5‐caffeoylquinic acid	19.10	336	181
7	p‐Coumaric	22.5	167	191
8	Ferulic acid	24.10	193	210

**TABLE 2:** Biochemical parameters in the serum of rats treated with aqueous extract of *Chamaerops humilis* (AECH).

Mean paw volume ± SEM (mL)					
Inhibition (%)					
	0 h	1 h	2 h	3 h	4 h
Control	1.60 ± 0.01	2.78 ± 0.02	2.50 ± 0.03	2.00 ± 0.02	3.94 ± 0.02
—	—	—	—	—	—
AECH	1.52 ± 0.01	2.30 ± 0.03	2.20 ± 0.04	1.97 ± 0.04	2.78 ± 0.04
250 mg/kg p.o.	—	22.0 ± 0.1	32.2 ± 0.01	20.0 ± 0.02	50.4 ± 0.01
AECH	1.51 ± 0.01	1.68 ± 0.08	1.95 ± 0.06	1.40 ± 0.08	1.90 ± 0.11
500 mg/kg p.o.	—	50.0 ± 0.01	60.7 ± 0.01	68.4 ± 0.008	87.0 ± 0.01
AECH	1.48 ± 0.01	1.50 ± 0.01	1.75 ± 0.06	1.20 ± 0.05	1.70 ± 0.08
1000 mg/kg p.o.	—	76.7 ± 0.008	50 ± 0.01	78.86 ± 0.003	95.8 ± 0.002
Indomethacin	1.55 ± 0.06	1.85 ± 0.03	1.98 ± 0.09	1.04 ± 0.10	2.55 ± 0.10
10 mg/kg p.o.	1.55 ± 0.06	96.7 ± 0.03	48 ± 0.01	95.67 ± 0.02	66.7 ± 0.01

Data are expressed as mean ± SEM.

### 2.2 Anti‐inflammatory Effect

#### 2.2.1 Xylene‐Induced Ear Edema in Mice

**FIGURE 1:**
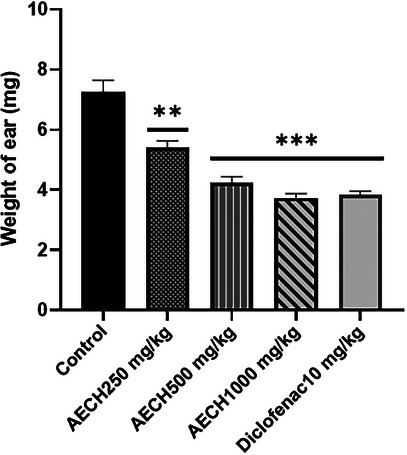
Anti‐inflammatory effect of AECH and diclofenac in Xylene‐Induced Ear edema test in mice. Data are presented as mean ± SEM (*n* = 6). **p < 0.001, ***p < 0.0001, a significant difference between treated groups and negative control. AECH: aqueous extract of *Chamaerops humilis* fruit.

The results in Figure 1 demonstrate a significant reduction in ear edema, reflected by the decreased weight of the right ear compared to the left (control) ear in mice following xylene application. Pretreatment with the AECH fruits at all doses produced a dose‐dependent reduction in ear weight. The effect was significant at 250 mg/kg (*p* < 0.001) and even more pronounced at 500 and 1000 mg/kg (*p* < 0.0001). Similarly, diclofenac significantly reduced ear edema.

#### 2.2.2 Histological Analysis

**FIGURE 2:**
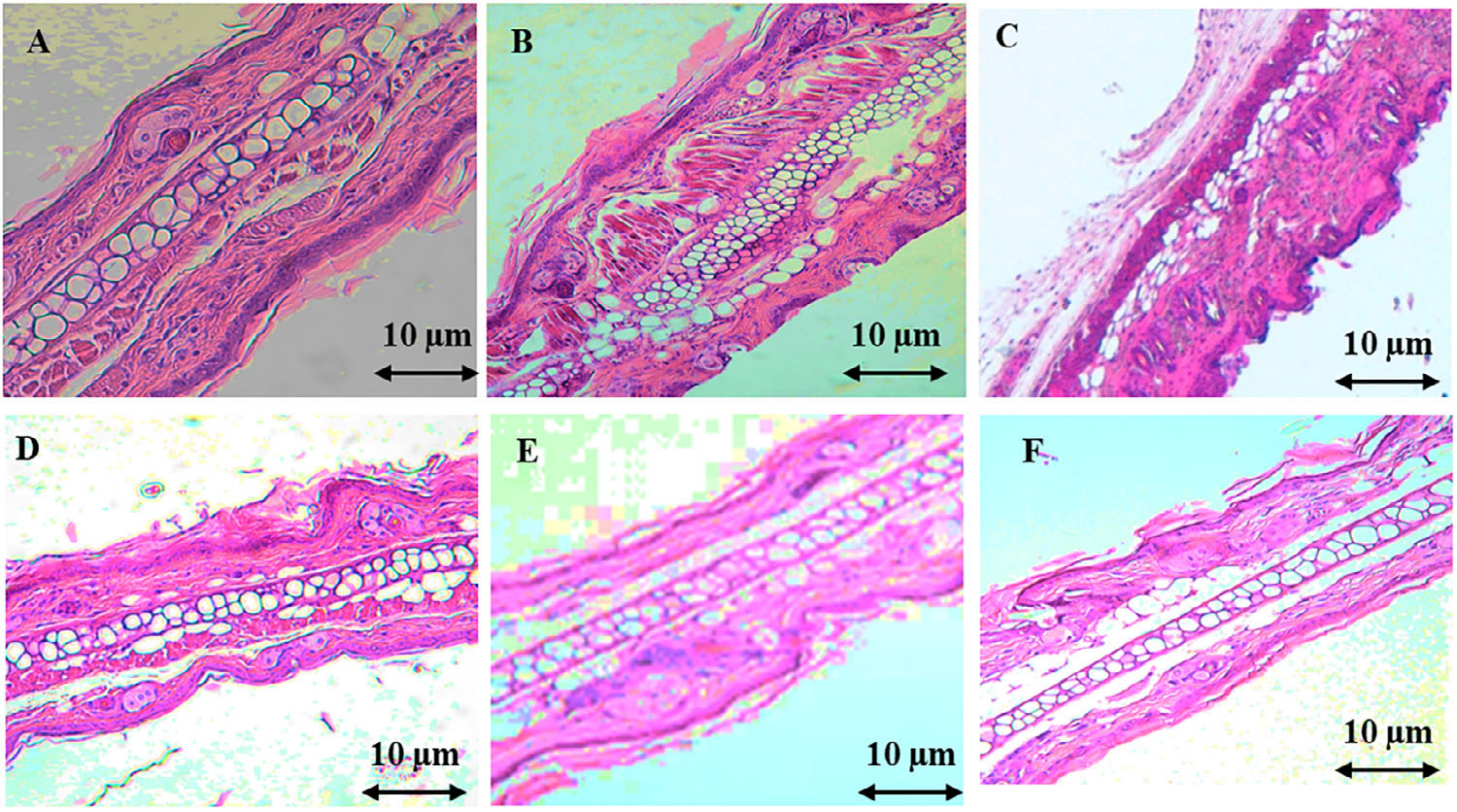
Histological sections of ear tissue illustrating swelling induced by xylene treatment. (A) Normal ear; (B) Model control group with xylene‐induced inflammation; (C) Indomethacin treatment (10 mg/kg); (D) aqueous extract of *Chamaerops humilis* (AECH) treatment at 250 mg/kg; (E) AECH treatment at 500 mg/kg; (F) AECH treatment at 1000 mg/kg. Sections were stained with H&E (×100). Scale bar = 10 µm.

Histological analysis of ear sections from mice with xylene‐induced edema revealed the infiltration of acute inflammatory cells, including neutrophils, plasma cells, and lymphocytes. The connective tissue stroma was heavily infiltrated with edema‐forming fluid, while the dermal lining remained intact. However, treatment with the AECH at doses of 500 and 1000 mg/kg demonstrated a notable anti‐inflammatory effect. This was evident at the cellular level by a reduction in fibrous connective tissue density and collagen content, while cartilage tissue appeared unaffected. In comparison, the reference drug indomethacin effectively reduced acute inflammatory cell infiltration, particularly in the sparse stromal region, and maintained the normal appearance of the epithelial nuclei in the dermal lining (Figure 2).

#### 2.2.3 Carrageenan‐Induced Rat‐Paw Edema

The findings presented in Table 2 show that intraplantar injection of carrageenan in rats led to a time‐dependent increase in paw volume, indicating the progression of edema. However, treatment with the AECH at doses of 250, 500, and 1000 mg/kg significantly reduced paw edema in a dose‐dependent manner at 1, 2, 3, and 4 h post‐carrageenan injection. Notably, the greatest reduction in paw volume was observed at the third hour, with a slight increase in edema by the fourth hour. Furthermore, the reference drug indomethacin (10 mg/kg) significantly (*p* < 0.001) inhibited carrageenan‐induced edema throughout the measurement period.

#### 2.2.4 Anti‐ulcer Effect on Rats

**FIGURE 3:**
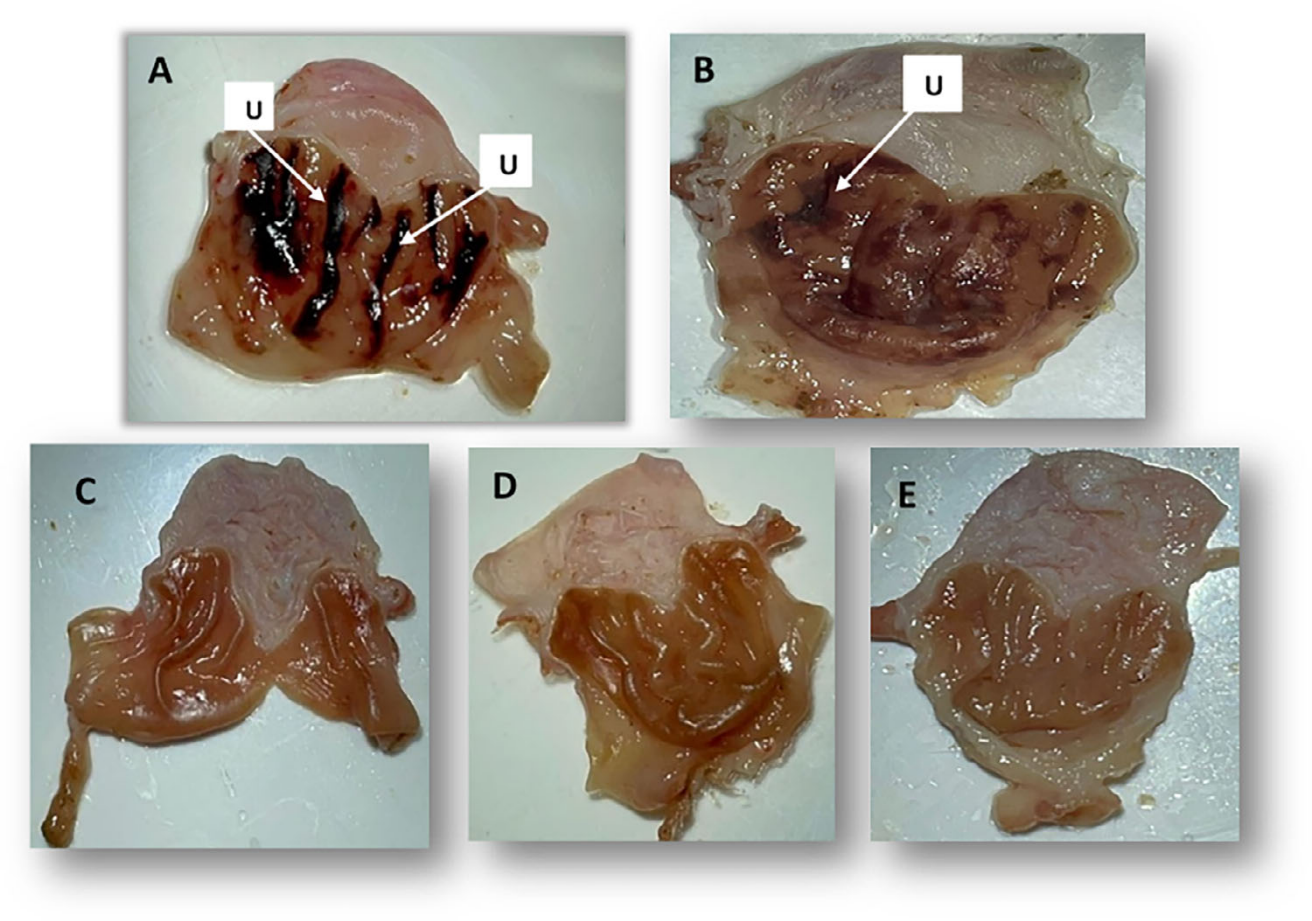
Photographs of gastritis caused by ethanol/hydrochloric acid (EtOH/HCL) solution in rat stomach. (A) Ulcerative rat (HCL/EtOH), (B) treated rat with AECH 250 mg/kg, (C) treated rat with AECH 500 mg/kg, (D) treated rat with AECH 1000 mg/kg, and (E) non‐ulcerative rat (Distilled water). (U: ulceration; AECH: Aqueous extract of *Chamaerops humilis* fruits).

The effects of AECH on EtOH/HCl‐induced gastric lesions are shown in Figure 3 and Table 3. Animals pretreated with AECH at 250 mg/kg exhibited minor lesions measuring 1.5 mm (Figure 2B) with a 66% inhibition of ulcers (Table 2). At higher doses of 500 and 1000 mg/kg, no macroscopic lesions were observed compared to the non‐ulcerative control group (Figure 2C–E), indicating 100% inhibition. In contrast, the ulcerative group showed severe gastric damage, with ulcer lesions measuring 3.5 mm. The reduction in lesions at all doses was statistically significant compared to the ulcerative group, with *p*‐values of <0.01 for the 250 mg/kg group and <0.001 for the 500 and 1000 mg/kg groups (Figure 3).

**TABLE 3:** Macroscopic parameters of gastric lesions induced by ethanol/hydrochloric acid (EtOH/HCL).

	US (mm)	UI	Score	I%
Control (non ulcerative rat)	0.00 ± 0.00	0.00 ± 0.00	0.00 ± 0.00	—
Ulcerative rat (HCL/EtOH) p.o.	2.5 ± 0.6***	3.1 ± 0.63***	1.5 ± 0.09***	—
AECH	1.1 ± 0.14^##^	0.5 ± 0.03^##^	2.20 ± 0.04**	66%
250 mg/kg p.o.				
AECH	0.00 ± 0.00^###^	0.00 ± 0.00^###^	1.95 ± 0.06***	100%
500 mg/kg p.o.				
AECH	0.00 ± 0.00^###^	0.00 ± 0.00^###^	1.75 ± 0.06***	100%
1000 mg/kg p.o.				

Values are presented as mean ± SEM. ***p < 0.001, Significant between non‐ulcerative rats and ulcerative rats. ^##^ *p* < 0.01; ^###^ *p* < 0.001, significant between treated rats and ulcerative rats (*n* = 6); US: ulceration surface; UI: ulcer index; US: ulceration surface UI: ulceration index I: inhibition percentage.

## 3 Discussion

The current study aimed to explore the anti‐inflammatory and anti‐ulcer properties of AECH using multiple experimental models, including carrageenan‐induced paw edema, xylene‐induced ear edema, and EtOH/HCl‐induced gastric ulcer. The results provide significant insights into the potential therapeutic benefits of AECH, particularly its anti‐inflammatory and gastroprotective properties.

The carrageenan‐induced paw edema test is a well‐established in vivo model commonly used to assess the anti‐inflammatory properties of compounds, particularly in the acute phase of inflammation [12]. In this study, AECH demonstrated a significant dose‐dependent reduction in paw edema at all measured time points (1, 2, 3, and 4 h post‐carrageenan injection) compared to the control group.

Carrageenan typically induces inflammation by stimulating the release of pro‐inflammatory mediators such as IL‐1β, IL‐6, COX‐2, and PGE2, which increase vascular permeability and promote tissue edema [13], The inflammatory process occurs in two distinct phases: the first phase (within 1–2 h) involves the release of histamine and serotonin, while the second phase is characterized by the release of leukotrienes and prostaglandins [12].

The observed reduction in paw volume, especially notable during the third hour, suggests that AECH may inhibit the later stages of the inflammatory response, likely by reducing the activity of prostaglandins and other inflammatory cytokines. The sustained and significant reduction in edema over time suggests that AECH exerts its effects by modulating both the early and late phases of inflammation. This aligns with the findings of Bellifa et al. [14], who noted that the anti‐inflammatory activity of *C. humilis* exceeds that of corticosteroids in a protein denaturation model.

Additionally, AECH exhibits significant antioxidant capacity and is rich in flavonoids, total polyphenols, and condensed tannins, underlining its potential as a therapeutic agent with multifaceted benefits. The presence of quinic acid, chlorogenic acid derivatives, and compounds like p‐coumaric and ferulic acids further enhances its therapeutic potential due to their antioxidant, anti‐inflammatory, and possible analgesic effects. Together, these findings suggest that AECH could be a valuable natural source for therapeutic applications, offering a broad spectrum of health benefits.

Similarly, in the xylene‐induced ear edema test, which is characterized by capillary dilation and increased vascular permeability, the topical application of xylene led to significant ear edema, as indicated by the increase in ear weight in the untreated control group [15]. However, pretreatment with AECH at doses of 250, 500, and 1000 mg/kg resulted in a dose‐dependent reduction of ear edema. This effect may be attributed to AECH's ability to inhibit the release of key pro‐inflammatory mediators, such as histamine and bradykinin, which are crucial to the inflammatory process. Supporting this, Miguel et al. [16] demonstrated that *C. humilis* extracts inhibit lipoxygenase (LOX), an enzyme central to inflammation. The presence of tannins and flavonoids in AECH, known for their free radical scavenging and anti‐inflammatory properties, likely contributes to these effects by reducing oxidative stress and modulating inflammation [17, 18, 19].

The results of the anti‐ulcer activity of AECH against HCl/EtOH‐induced ulcers in rats indicate that this extract has a notable antiulcerogenic potential, providing significant gastroprotective effects. At both tested doses, AECH exerted a dose‐dependent cytoprotective action, as evidenced by the reduction in ulcer surface area and an increase in the percentage of ulcer inhibition achieving 100% at the highest dose.

The HCl/EtOH solution is a well‐known gastro‐toxic agent that decreases gastric mucus and severely damages the stomach's cell membrane [20]. Ethanol penetrates the mucosal layer down to the submucosa, leading to lesions such as erosion, hemorrhage, and ulcers [21]. The protective effect of AECH may be attributed to several mechanisms of action. AECH enhances gastric mucosal defense by stimulating mucus secretion, which forms a crucial barrier that protects the stomach lining from aggressive agents like HCl and EtOH. Additionally, AECH's rich composition of bioactive compounds, including flavonoids (such as rutin and kaempferol) and phenolic acids (like chlorogenic and ferulic acid), provides strong antioxidant properties [22, 23]. These compounds have been found to effectively scavenge reactive oxygen species generated during oxidative stress, thereby mitigating oxidative damage to the gastric mucosa and reducing cell apoptosis [24]. Moreover, AECH may inhibit the activity of enzymes involved in ulcerogenesis, including COX and LOX, which are responsible for synthesizing pro‐inflammatory mediators like prostaglandins and leukotrienes. By inhibiting these pathways, AECH not only reduces inflammation but also enhances the production of cytoprotective prostaglandins that are vital for maintaining mucosal integrity. We acknowledge that the evaluation of the anti‐inflammatory and gastroprotective effects in this study primarily relies on histological and macroscopic observations, which may have certain limitations. To strengthen the rigor of our findings, it would be valuable to include inflammatory biomarkers, such as pro‐inflammatory cytokines or inflammation‐related proteins, in future studies to complement the histological analysis. These biomarkers would allow for more precise quantification of the biological effects of the treatment and provide additional validation of the observed effects through more targeted and molecular approaches. Furthermore, incorporating biomolecular studies and deeper analyses of underlying cellular mechanisms would enhance the understanding of the processes involved and offer more robust validation of the anti‐inflammatory and gastroprotective effects.

## 4 Conclusions

In summary, this study highlights the significant anti‐inflammatory and anti‐ulcer effects of the AECH. AECH reduces inflammation and enhances gastric mucosal defense through its bioactive compounds, such as flavonoids and phenolic acids. These findings confirm its traditional use as a natural remedy for inflammatory conditions and gastric ulcers, suggesting that AECH may serve as a promising therapeutic agent warranting further research into its potential applications.

## 5 Experimental

### 5.1 Plant Samples

The plant was collected in 2021 in Azilal, a region located in the central part of the Atlas Mountains in Morocco (31°57′41″ N, 6°34′15″W). The plant material, consisting of fruits, was cleaned and shade‐dried at room temperature (25°C) before undergoing extraction.

The extract was prepared using the subsequent method: the plant materiel was crushed until it formed a powder. Then, 30 g of dried fruits were stirred in 300 mL of distilled water for 12 h under agitation at room temperature (22°C ± 2°C), followed by a more delicate filtration on Whatman filter paper. The resulting filtrate was frozen and lyophilized. Finally, the powder was stored at –20°C in a desiccant until needed. The yield of the extract was 16.66%.

### 5.2 LC–MS/MS Analysis

The phytochemical analysis of the AECH was conducted via HPLC‐PDA‐MS/MS. A SHIMADZU LC‐MS 8050 system (Shimadzu, Japan), equipped with a triple quadrupole spectrometer with an electrospray ionization source, was used. Separation was achieved using a C18 reversed‐phase column (Zorbax Eclipse XDB‐C18, rapid resolution, 4.6 × 150 mm, 3.5 µm; Agilent, Santa Clara, CA, USA). The elution was performed with a gradient of water and acetonitrile (ACN), each containing 0.1% formic acid, progressing from 5% to 30% ACN over 60 min at a flow rate of 1 mL/min. Sample injection was automated via a SIL‐40C xs autosampler. The LC solution software (Shimadzu, Japan) controlled the instrumentation, and the MS operated in negative ion mode. For compound identification, mass spectra obtained by fragmentation were compared with those available in the literature and referred to as pure compounds. No reference standards were used.

### 5.3 Animals

Male and female Sprague Dawley rats and Swiss albino mice were used in this study weighing respectively (150–200 g) and (20–32 g) were obtained from the animal house of the Faculty of Sciences Semlalia, Marrakech, Morocco. They were acclimatized before proceeding and kept under standard conditions and kept in standard laboratory conditions, with a 12‐h light/dark cycle, a temperature of (20°C ± 2°C) and about 24%–50% relative humidity. Throughout the study, the mice and rats were treated in accordance with the international guidelines for the care and use of animals in research.

All the animals were separated into five groups (*n* = 6) according to the following design.

Development of an anti‐inflammatory model

Ear edema model (mice)

Group 1: Negative control (Tween 80, NaCl 0.9 %, p.o.)

Group 2: xylene‐induced ear edema + *C. humilis* (250 mg/kg, p.o.)

Group 3: xylene‐induced ear edema + *C. humilis* (500 mg/kg, p.o.)

Group 4: xylene‐induced ear edema + *C. humilis* (1000 mg/kg, p.o.)

Group 5: Positive control xylene‐induced ear edema + Diclofenac sodium (10 mg/kg i.p.)

Paw edema model (rats)

Group 1: Negative control of inflammatory assay (sodium chloride (Tween 80, NaCl 0.9 %, p.o.)

Group 2: carrageenan‐induced paw edema + *C. humilis* (250 mg/kg, p.o.)

Group 3: carrageenan‐induced paw edema + *C. humilis* (500 mg/kg, p.o.)

Group 4: carrageenan‐induced paw edema + *C. humilis* (1000 mg/kg, p.o.)

Group 5: carrageenan‐induced paw edema + Indomethacin (IND) (10 mg/kg, p.o.)

Development of ulcer model (rats)

Group 1: Negative control of anti‐ulcer activity (NaCl 0.9 %)

Group 2: gastric ulcer induced by Hydrochloric acid (HCL)/Ethanol mixed + *C. humilis* 250 mg/kg)

Group 3: gastric ulcer induced by HCL/Ethanol mixed + *C. humilis* (500 mg/kg)

Group 4: gastric ulcer induced by HCL/Ethanol mixed + *C. humilis* (1000 mg/kg)

Group 5: Positive control of anti‐ulcer activity (0.8 M HCl in 60% ethanol p.o.)

### 5.4 Drugs and Treatments

Xylene and carrageenan were obtained from Sigma Aldrich (St. Louis, MI, USA). IND was administered intraperitoneally at a dose of 10 mg/kg. Diclofenac sodium was administered at a dose of 10 mg/kg orally. A mixture of HCl solution (0.8 M) and ethanol solution (60%) was administered orally. The negative control group was treated with the vehicle Tween 80 (10 mL/kg) +0.9%. Doses (250, 500, and 1000 mg/kg) of AECH are administered orally.

### 5.5 Anti‐inflammatory Effect

#### 5.5.1 Xylene‐Induced Ear Edema in Mice

The ear edema was induced using a method previously described by Akindele & Adeyemi [25]. Each group of mice (*n* = 6/group) was administered a single oral dose of *C. humilis* extract (250, 500, and 1000 mg/kg) 30 minutes before the procedure, while the control group received physiological saline. Diclofenac sodium (10 mg/kg) served as the positive control. The left ear, which received no treatment, was used as a control, and 20 µL of xylene was topically applied to the anterior and posterior surfaces of each mouse's right ear lobe. One hour after the xylene application, the animals (six per group) were humanely sacrificed by cervical dislocation in compliance with ethical guidelines. The right and left ears were excised, and 5 mm sections were collected and weighed. Ear edema was evaluated by determining the weight difference between the corresponding sections of the right and left ears of each animal. Tissue samples were then fixed in 10% formalin, sectioned at a thickness of 4 µm, and stained with hematoxylin and eosin for histological analysis.

The percentage of edema inhibition was calculated according to the following relationship:

$$\begin{array}{ccc} \mathrm{inhibition}\left(\%\right) & = & \;\text{difference in ear weight}\;\left(\mathrm{control}\right)/\\ & & \text{difference in ear weight}\;\left(\text{treat}\right)/\\ & & \text{difference in ear weight}\;\left(\text{control}\right)\times 100\% \end{array}$$

#### 5.5.2 Carrageenan‐induced Rat‐Paw Edema

Following the protocol described by Winter et al. [26], with some modifications, male Sprague–Dawley rats (n = 6/group), weighing between 150 and 200 g, were fasted for 18 h prior to the experiment. The rats were divided into groups and administered AECH at doses of 250, 500, and 1000 mg/kg, or indomethacin at a dose of 10 mg/kg, which served as the positive control. The volume of the right hind paw of each rat was measured in milliliters before the induction of edema. Following this, 0.1 mL of carrageenan (1% suspension in 0.9% saline) was injected into the subplantar area, and paw volume was subsequently measured every hour over a 4‐h period [26].

Paw volume was assessed by immersing the paw in distilled water contained in a 100 mL beaker, with the displaced water volume corresponding to the paw size. To ensure precise and consistent measurements, each rat's paw was marked.

The degree of edema was calculated by comparing the increase in paw volume after carrageenan injection to the baseline volume measured before injection for each rat. The results were expressed as the percentage of edema inhibition in the treated groups compared to the control group.

The evaluation of anti‐inflammatory activity was performed by calculating the percentage of edema inhibition in the presence of the extract using the following formula:

$$\begin{array}{ccc} \%\mathrm{inhibition} & = & \left[\left(\mathrm{Vn}-\mathrm{Vo}\right)\mathrm{control}-\left(\mathrm{Vn}-\mathrm{Vo}\right)\mathrm{treated}\right]/\\ & & \left(\mathrm{Vn}-\mathrm{Vo}\right)\mathrm{control}\times 100 \end{array}$$

The percentage increase in edema was calculated using the following formula:

$$\%\;\text{increase in edema}\;=\left[\left(\mathrm{Vn}-\mathrm{Vo}\right)/\mathrm{Vo}\right]\times 100$$

where Vn is the average volume measured at each hour over the 4‐h period following the carrageenan injection, and Vo is the average volume measured before the injection.

### 5.6 Anti‐ulcer Activity in Rats

The ulcer was induced according to the method of Hara et Okabe [27]. The rats of either sex (*n* = 6/group) were fasted for 24 h. One hour before ulcer induction, the control group received orally 1 mL of distilled water, and the test groups received the AECH at doses 250, 500, and 1000 mg/kg). Gastric lesion was induced by the administration of HCl/EtOH mixture (0.8 M HCl in 60% ethanol at a dose of 1 mL/kg. One hour after ulcer induction the animals were ether‐anesthetized, sacrificed by cervical dislocation, and then the stomach was removed and incised along the axis of greater curvature and washed with distilled water. The dimension of the ulcer was measured with a planimeter [27].

The evaluation of anti‐ulcer activity was performed by calculating the lesions measured, and the scores according to the method of Tan et al. [28].

Lesion Scores:

- Grade 1: lesion ≤1 mm
- Grade 2: lesion between 1 and 2 mm
- Grade 3: lesion ≥2 mm

Ulcer Index Calculation Formula:

$$\begin{array}{ccc} \mathrm{UI} & = & (1\times \left(\text{number of grade}\;1\mathrm{lesions}\right)\\ & & +\;2\times \left(\text{number of grade}\;2\;\text{lesions}\right)\\ & & +\;3\times \left(\text{number of grade}\;3\;\text{lesions}\right))/10 \end{array}$$

Percentage Inhibition Formula:

$$\begin{array}{ccc} \%I & = & (1-\;\text{ulcer index of the test group/}\\ & & \text{ulcer index of the control group})\times 100 \end{array}$$

### 5.7 Statistical Analysis

The findings were presented as the mean ± standard error of the mean and assessed using GraphPad Prism 10.2.3 statistical software, data analysis involved conducting one‐way analysis of variance. Statistical significance was determined when the *p*‐value was below 0.05 (*p* < 0.05) for all evaluations. This comprehensive approach ensured the validity of the parametric analysis methods used in this study.

## Author Contributions

**Yasmina Jaouhari**: conceptualization, methodology, validation, investigation, writing – original draft preparation, writing – review and editing. **Hamid Kabdy**: conceptualization, methodology. **Ouijdane El hatimy**: conceptualization^.^ **Hajar Azraida**: methodology, writing – original draft preparation. **Yassine Chait**: writing – original draft preparation. **Rachida Aboufatima**: writing – review and editing. **Soad Moubtakir**: writing – review and editing, supervision. **Loubna El Yazouli**: investigation and writing – review and editing, supervision. **Stefania Garzoli**: validation, writing‐review and editing, supervision. **Abderrahman Chait**: methodology, investigation, supervision. All authors contributed to editorial changes in the manuscript. All authors read and approved the final manuscript. All authors have participated sufficiently in the work and agreed to be accountable for all aspects of the work.

## Conflicts of Interest

The authors declare no conflicts of interest.

## Ethics Statement

All experiments were conducted in compliance with the guidelines of the European Community (Directive 86/609/EEC, November 24, 1986). The animal study protocol was approved by the Institutional Review Board of the Faculty of Sciences at Cadi Ayyad University in Marrakech, Morocco (protocol code KH00‐287/01/23, approval date February 2024). Every effort was made to minimize the number of animals used in all experiments.

## Data Availability

The authors have nothing to report.
